# Low insulin-like growth factor-1 level predicts survival in humans with exceptional longevity

**DOI:** 10.1111/acel.12213

**Published:** 2014-03-12

**Authors:** Sofiya Milman, Gil Atzmon, Derek M Huffman, Junxiang Wan, Jill P Crandall, Pinchas Cohen, Nir Barzilai

**Affiliations:** 1 Department of Medicine, Division of Endocrinology, Albert Einstein College of Medicine1300 Morris Park Ave, Bronx, NY, USA; 2 Institute for Aging Research, Albert Einstein College of Medicine1300 Morris Park Ave, Bronx, NY, USA; 3 Department of Genetics, Albert Einstein College of Medicine1300 Morris Park Ave, Bronx, NY, USA; 4 Department of Molecular Pharmacology, Albert Einstein College of Medicine1300 Morris Park Ave, Bronx, NY, 10461, USA; 5 Davis School of Gerontology, University of Southern California3715 McClintock Avenue, Los Angeles, CA, 90089, USA

**Keywords:** IGF-1, insulin-like growth factor 1, mortality, longevity, human, cancer

## Abstract

Attenuated growth hormone and insulin-like growth factor-1 (GH/IGF-1) signaling is associated with extended lifespan in several animal models. However, the effect of diminished GH/IGF-1 activity on survival in humans has not been confirmed. We tested the hypothesis that IGF-1 levels in nonagenarians (*n* = 184), measured at study enrollment, predict the duration of their incremental survival. In the Kaplan–Meier analysis, females with IGF-1 levels below the median (≤ 96 ng mL^−1^) had significantly longer survival compared with females with levels above the median, *P* < 0.01. However, this survival advantage was not observed in males (*P* = 0.83). On the other hand, in both males and females with a history of cancer, lower IGF-1 levels predicted longer survival (*P* < 0.01). IGF-1 level remained a significant predictor of survival duration in linear regression models after multivariable adjustment in females (*P* = 0.01) and individuals with a history of cancer (*P* < 0.01). We show for the first time that low IGF-1 levels predict life expectancy in exceptionally long-lived individuals.

Diminished growth hormone/insulin-like growth factor-1 (GH/IGF-1) signaling has been linked to extended survival in several animal species (Kenyon *et al*., 1993; Brown-Borg *et al*., 1996). However, the role of the GH/IGF-1 axis in human survival and disease remains inconclusive. Higher IGF-1 levels have been associated with lower incidence of cardiovascular disease (CVD) and cognitive dysfunction in the general population (Sonntag *et al*., 2012), but lower IGF-1 levels have been related to decreased incidence of several forms of cancer (Renehan *et al*., 2004). Furthermore, individuals with absent GH receptor appear to be protected from developing cancer and type 2 diabetes (T2DM; Guevara-Aguirre *et al*., 2011).

Individuals with exceptional longevity comprise an advantageous group for the study of mechanisms that promote healthy aging, as many of them have delayed onset or have been spared from age-related diseases (Andersen *et al*., 2012). Diminished IGF-1 signaling may be one such mechanism. Our group showed that a functional mutation in the IGF-1 receptor, which confers partial IGF-1 resistance, was more prevalent in centenarians, as compared to controls without familial longevity (Suh *et al*., 2008). Based on these observations in humans and other species, we hypothesized that lower IGF-1 levels are predictive of extended survival in generally healthy nonagenarians.

Subject characteristics at the time of IGF-1 measurement are listed in Table 1. The survival time, as depicted by the Kaplan–Meier curves, was significantly longer in females with IGF-1 levels below the median, compared with females with IGF-1 above the median, *P* < 0.01 (Fig. 1A). However, this survival advantage was not observed in males (*P* = 0.83, Fig. 1B). There was a no significant association between IGF-1 levels and survival among individuals without a history of malignancy (Fig. 1C). Females without malignancy and with low IGF-1 exhibited a trend toward longer survival. On the other hand, in the group with a history of malignancy, individuals with low IGF-1 levels had significantly prolonged survival compared with individuals with high IGF-1 levels (*P* < 0.01, Fig. 1D). Among the subjects with a history of cancer, the median survival was 49.6 months in the group with low IGF-1 and 20.7 months in the group with a high IGF-1 (*P* < 0.01).

**Table 1:** Subject characteristics according to IGF-1 groups† (*n* = 184)

Characteristic	Low IGF-1 (*n* = 93)	High IGF-1 (*n* = 91)	*P*-value
IGF-1, ng mL^−1^, median (IQR)	55 (36–72)	121 (112–158)	< 0.001
Age, years, median (IQR)	96.8 (95.9–98.1)	96.7 (95.6–97.9)	0.40
Sex, female%	73.1	78.0	0.44
Height‡, in (*n* = 135)	63.9 ± 3.3	63.2 ± 3.4	0.26
BMI§, kg m^−2^ (*n* = 126)	21.5 ± 3.6	21.6 ± 3.2	0.86
HDL, mg dL^−1^, mean ± SD	53.1 ± 14.8	52.5 ± 17.3	0.81
Glucose, mg dL^−1^ (*n* = 179)	102.2 ± 33.1	113.4 ± 43.2	0.05
Insulin, µU mL^−1^, median (IQR) (*n* = 81)	19.9 (10.7–35.7)	26.5 (11–44.2)	0.30
HOMA-IR, median (IQR) (*n* = 81)	4.9 (2.4–8.5)	7.1 (2.4–13.5)	0.20
Cancer¶, % (*n* = 151)	20.3 (15)	24.7 (19)	0.52
Diabetes mellitus, % (*n* = 142)	5.6 (4)	8.5 (6)	0.75
Cardiovascular disease,% (n=141)	22.5 (16)	27.1 (19)	0.53
Cognitive impairment**, % (*n* = 171)	52.4 (44)	49.4 (43)	0.70

† Groups dichotomized at the median IGF-1 level, 96 ng mL^−1^.

‡ Maximum adult height.

§ BMI calculated using the maximum adult height.

¶ Cancer diagnoses exclude nonmelanoma skin cancer.

** Defined as Mini-Mental Examination (MMSE) score < 25 or Blind MMSE score < 16.

**Figure 1:**
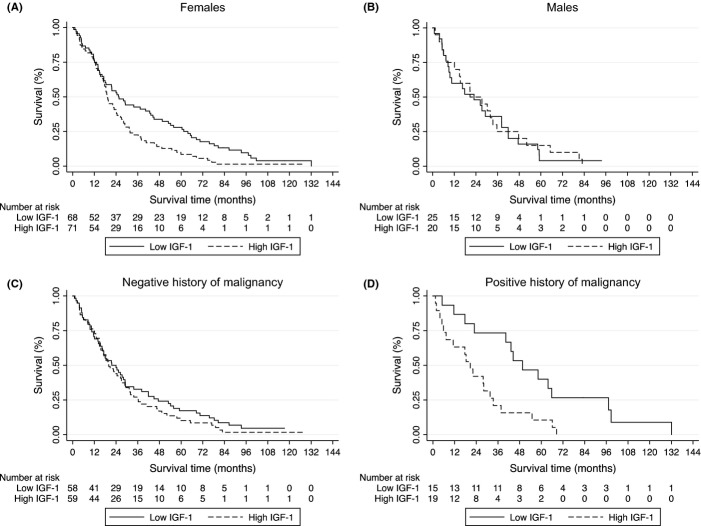
Kaplan–Meier curves for groups with IGF-1 levels below (low IGF-1) and above (high IGF-1) the median (A–D). *P*-values for comparison between IGF-1 groups. (A) Females, *P* < 0.01. (B) Males, *P* = 0.83. (C) Negative history of malignancy, *P* = 0.42. (D) Positive history of malignancy, *P* < 0.01.

Sex-stratified linear regression models, adjusted for age, high-density lipoprotein cholesterol (HDL), CVD, and T2DM, confirmed that IGF-1 was independently associated with survival only in females (*P* = 0.01 in females and *P* = 0.89 in males). In women, each 1 ng mL^−1^ increase in IGF-1 was associated with an average (95% CI) decrease of 0.1 (−0.18 to −0.02) months in survival. This model significantly predicted survival time in females, *P* < 0.01, and IGF-1 explained approximately 6.2% of the variability in survival.

After stratification of all subjects by a history of cancer, IGF-1 was inversely associated with survival duration only in individuals with a positive history of malignancy, after adjustment for age, sex, HDL cholesterol, CVD and T2DM (*P* < 0.01). In this group, each 1 ng mL^−1^ rise in IGF-1 level was related to a mean decline of 0.27 (−0.45 to −0.09) months in survival, *P* < 0.01. This model significantly predicted survival time (*P* = 0.01), with 23% of the variability in survival attributed to IGF-1. Additional adjustments for glucose and insulin levels did not modify these associations.

Reports linking IGF-1 levels with mortality in older adults have been inconsistent (Roubenoff *et al*., 2003; Kaplan *et al*., 2012). We have demonstrated in relatively healthy nonagenarians that lower IGF-1 levels significantly predict survival, specifically in females and individuals with a history of cancer. This finding provides evidence that the diminution of the GH/IGF-1 axis may extend human survival.

The gender-specific effect of lower IGF-1 on extended survival was noted in other species. Several, though not all, rodent models with attenuated GH and insulin/IGF-1 signaling exhibited improved longevity in females only (Sonntag *et al*., 2012). Whether the female-preferential result of this study is due to interaction with factors unique to the female hormonal milieu or to other sex differences in tissue and cellular function remains unknown and presents an important question for investigation.

The longer survival time among subjects with low IGF-1 levels was most notable in those with a history of malignancy. In rodents with attenuated GH/IGF-1 signaling, a decreased incidence of malignancy was also observed (Ikeno *et al*., 2003). Indeed, IGF-1 is a potent stimulator of cell growth and proliferation and has been associated with increased risk of several types of cancers in animals and humans (Renehan *et al*., 2004). Thus, it is possible that the observed beneficial effect of low IGF-1 levels on survival in humans is predominantly a mechanism for extended survival in individuals susceptible to malignant proliferation. As cancer risk rises exponentially with age and is the second leading cause of mortality in adult US population (CDC/NCHS National Vital Statistics System, 2010), reducing its incidence through inhibition of IGF-1 action may have a major impact on morbidity and mortality in the elderly.

Populations with longevity are advantageous for investigation of factors that inhibit disease development or progression and promote survival. Although this study cannot delineate whether low IGF-1 levels existed earlier in the life course of these individuals, there is some evidence linking longevity and lower prevalence of several diseases with a reduction in the GH/IGF-1 signaling throughout the lifespan (Suh *et al*., 2008; Guevara-Aguirre *et al*., 2011). Thus, we conclude that attenuation of the GH/IGF-1 axis may play an important role in extending survival in humans who achieve exceptional longevity, although this effect may be gender and disease specific. Furthermore, our results provide additional evidence against the rationale for treating older adults with GH replacement as an ‘antiaging’ strategy.

## Supporting Information

**Supplement file** Experimental procedures.
