# Supplementary material for: Low insulin-like growth factor-1 level predicts survival in humans with exceptional longevity
Source: Aging Cell. 2014 Mar 12;13(4):769–71. doi: 10.1111/acel.12213 (PMC4116456; doi:10.1111/acel.12213)
Supplement: Supplementary file 1 — Supplement file Experimental procedures. [file acel0013-0769-sd1.docx]

**Supporting Information:**

**Experimental Procedures:**

Ashkenazi Jews, age 95 years or older, n=553, were recruited for the Longevity Genes Project at the Albert Einstein College of Medicine, from the Northeastern United States between 1998 to the present, as previously described ([Atzmon *et al.* 2004](#_ENREF_2)). This population originated from a small number of founders and remained relatively homogeneous genetically and in its socioeconomic status. Eligible participants were living independently at age 95 years, as a reflection of good health; although, at recruitment they may have been at any level of dependency.

At enrollment, a study nurse visited each participant in his or her residence and obtained a thorough medical history using a structured questionnaire, performed a physical assessment, and collected a fasting venous blood sample. Serum glucose, insulin and HDL cholesterol were measured at the time of enrollment, by standard automated methods at the clinical laboratories of Montefiore Medical Center, Bronx, NY. Homeostatic model assessment-insulin resistance ([Matthews *et al.* 1985](#_ENREF_9)) (HOMA) was calculated using the formula: (glucose x insulin)/405. The remainder of the blood sample was processed and serum was stored at -80°C. Serum levels of human IGF-I were measured in stored samples in a subgroup of participants recruited prior to 2008 by specific in-house ELISA. Recombinant human IGF-I standards, monoclonal antibodies and biotinylated polyclonal antibodies were purchased from R&D Systems (Minneapolis, MN). The IGF-I assay has a sensitivity of 0.1 ng/mL and intra- and inter-assay CVs are 6% and 8%, respectively. Prior to human IGF-I assays, serum samples were extracted with acid/ethanol. The absorbance was measured on a plate spectrophotometer (Molecular Designs, Sunnyvale, CA) at 490 nm. Individuals with previously identified functional IGF-1 receptor mutations (Ala-37-Thr and Arg-407-His) ([Suh *et al.* 2008](#_ENREF_13)) were excluded from the present analysis, as this mutation may alter IGF-1 levels and normal IGF-1 physiology.

IGF-1 levels and survival time were available for 184 participants. Survival duration was calculated as the number of months the participant lived after enrollment into the study. The date of death was obtained by searching a national death database, referencing the participants’ names and social security numbers. For the 5 subjects who were alive at the time of analysis, survival duration was calculated as the number of months that the participants survived from the date of study enrollment until the date of analysis and the subjects were censored after that time. Disease prevalence was self-reported and was derived from the questionnaire. For this analysis, cancer was defined as any malignancy, with the exclusion of non-melanoma skin cancer. However, the format of the questionnaire did not distinguish between active malignant disease and malignant disease that was in remission or cured. T2DM was defined by a history of diagnosed T2DM or taking medications for the treatment of T2DM. CVD was defined as having a history of at least one of the following: myocardial infarction, cerebrovascular event, percutaneous coronary intervention or coronary artery bypass graft surgery. Cognitive impairment was defined by a Mini-Mental Status Examination (MMSE) score of <25 for visually intact individuals or a score of <16 on the Blind MMSE in individuals with severe visual deficit. Written informed consent was obtained from the participants or their proxies, in the event that the subject lacked cognitive capacity. The study was approved by the Institutional Review Board at the Albert Einstein College of Medicine.

Statistical analysis was performed using STATA software, version 12 (StataCorp LP, College Station, TX). Normality was assessed by inspection of the histograms. Subject characteristics were compared using bivariate statistics, with non-parametric tests applied when appropriate. The Chi square statistic was used to compare categorical variables. Correlations were evaluated with Spearman’s rank correlation, due to non-parametric distribution of the variables of interest. Survival was graphed using the Kaplan-Meier curves and survival was compared between groups using the log-rank statistic. IGF-1 groups were dichotomized at the median IGF-1 level (96 ng/mL) for survival analysis and IGF-1 was entered as a continuous variable in the linear regression models. A non-automated forward building process was used for the linear regression models, with duration of survival as the dependent variable. Linear regression models excluded the 5 censored subjects, who were still living at the time of the analysis. Models were adjusted for variables determined to be confounders or considered to be meaningful predictors of survival. First order multiplicative interactions were assessed and were found to be present between IGF-1 and sex, as well as, between IGF-1 and cancer, excluding non-melanoma skin cancer. Therefore, final models were stratified by gender and history of Parametric data is presented as mean ± standard deviation (SD) and nonparametric data as median (interquartile range). A p-value <0.05 was considered to be statistically significant.

**Supporting Information References:**

Atzmon G, Schechter C, Greiner W, Davidson D, Rennert G , Barzilai N (2004). Clinical phenotype of families with longevity. *J Am Geriatr Soc*. **52**, 274-277.

Matthews DR, Hosker JP, Rudenski AS, Naylor BA, Treacher DF , Turner RC (1985). Homeostasis model assessment: insulin resistance and beta-cell function from fasting plasma glucose and insulin concentrations in man. *Diabetologia*. **28**, 412-419.
